# Evidence of carbon-supported porphyrins pyrolyzed for the oxygen reduction reaction keeping integrity

**DOI:** 10.1038/s41598-022-11820-6

**Published:** 2022-05-16

**Authors:** Walter Orellana, César Zúñiga Loyola, José F. Marco, Federico Tasca

**Affiliations:** 1 Departamento de Ciencias Físicas, Universidad Andrés Bello, Sazié 2212, 837-0136 Santiago, Chile; 2 Departamento de Química de Los Materiales, Facultad de Química y Biología, Universidad de Santiago de Chile, Santiago, Chile; 3 Instituto de Química Física “Rocasolano” CSIC, Madrid, Spain

**Keywords:** Electrocatalysis, Fuel cells

## Abstract

Fe(III) 5,10,15,20-(tetraphenyl)porphyrin chloride (FeTPP) and Co(III) 5,10,15,20-(tetraphenyl)porphyrin chloride (CoTPP) were adsorbed on carbon Vulcan and studied as electrocatalysts for the oxygen reduction reaction (ORR) before and after pyrolysis. The pyrolysis process was also simulated through ab initio molecular dynamic simulations and the minimum energy path for the O_2_ dissociation after the interaction with the metal center of the FeTPP and CoTPP were calculated. After the pyrolysis the FeTPP showed the best performances reducing O_2_ completely to H_2_O with increased limiting current and lower overpotential. Tafel slops for the various catalysts did not change after the pyrolytic process suggesting that the mechanism for the ORR is not affected by the heat treatment. TEM images, X-ray diffraction, XPS spectroscopy, ^57^Fe Mössbauer, and DFT simulations, suggest that there is no breakdown of the macrocyclic complex at elevated temperatures, and that the macro cyclic geometry is preserved. Small variations in the Metal-O_2_ (M-O_2_) binding energies and the M–N bond length were observed which is attributed to the dispersive interaction between the macrocycles and the irregular surface of the Vulcan substrate induced by the heat treatment and causing better interaction with the O_2_ molecule. The theoretical strategy herein applied well simulate and explain the nature of the M–N–C active sites and the performances towards the ORR.

## Introduction

Several plans of action are under study to decrease the environmental burden caused by the accumulation of greenhouse gases^1^. Low-temperature fuel cells emerge as a promising strategy to this proposal^2,3^. The H_2_ oxidation at the anode and the O_2_ reduction reaction (ORR) at the cathode would allow to secure up to four-electrons with a theoretical potential of 1.23 V and to obtain water as by-product. The cathodic reaction is the rate limiting step which is hampering the mass diffusion of this technology because it is too slow in alkaline media and unstable at low pHs. Platinum and Pt-based catalysts accelerate the reaction to appreciable paces^4,5^, but the price and scarcity of this noble metal are the limits^6^. Transition metals nitrogen complexes (MN4) have been studied as a possible replacement for the Pt-metal group since 1964, when Jasinsky reported that Co phthalocyanine (CoMN4) had catalytic activity for the ORR^7^. But MN4 complexes show lower performances than the Pt catalysts and no-stability in acid^8–10^. To increase the catalytic activities several strategies have been devised. Among them: (*i*) the use of carbon-based nanomaterial as supporting matrix to increase surface area, and conductivity^11–15^; (*ii*) the shift of the metal redox center (M^n+1/n^) to more positive values with electron-withdrawing peripherical substituents, so to increase the onset potential (*E*_onset_) for the ORR according to volcano correlation^10,16–19^; (*iii*) the pyrolysis of MN4 to modify the N-metal bond length (M–N–C) and the interaction of the metal center with O_2_^20–25^ (M-O_2_). Jahnke and co-workers were the pioneers of pyrolyzing complexes, with a seminal work in 1976^26^. Later developments have seen the use of metal-salts (Co, Fe, Mn) and N–C matrices as independent precursors to obtain active pyrolyzed catalysts for the ORR^27–35^.

After heat treatment of MN4 catalysts, the identification of the M–N–C active site is a crucial step to obtain insights into the ORR mechanism. Spectroscopic technics as Mössbauer, XPS, EXAFS, and XAS^20,24,36–41^, are powerful tools to define the M-N_x_ coordination nature. Density functional theory (DFT) calculations have been developed to study the interactions between the N atoms and the metal centers of the catalyst and to obtain the binding energy (E_bin_) as thermodynamic descriptor for the ORR^42–44^. When integrated with spectroscopic and electrochemical experiments, DFT simulations, can merge experimental results granting comprehensive perspectives^32,45,46^. It is a general agreement that MN_x_, and MN_x+1_ (where x: 2 + 2 or 4, and M is Co or Fe) are the most M–N–C active sites for the ORR where the formation is the result of the insertion of the metal into the carbon matrix^28,37,38,47,48^. However, when the MN4 complex is supported on carbon matrix, and then pyrolyzed, the nature of the active site is still under debate. Some authors claim that the macrocycle is broken up at low pyrolysis temperatures, (~ 800 °C, which is also considered as the optimal pyrolysis temperature to obtain electrocatalysts^49–51^), and then the MN4 active site is inserted into the carbon matrix, analogously to others M–N–C formed by independent precursors^21,24,52^. Other research groups suggest that the macrocyclic structures do not change after the heat treatment and remain adsorbed on the carbon support or that only the peripheral substituents of the MN4 are removed^53–55^.

To obtain more insights into the electrocatalytic active sites of pyrolyzed MN4 complexes, we performed ab *initio* molecular dynamics to simulate the pyrolysis process of Fe(III) 5,10,15,20-(tetraphenyl)porphyrin chloride (FeTPP) and Co(III) 5,10,15,20-(tetraphenyl)porphyrin chloride (CoTPP). We correlated the simulated results to spectroscopic (TEM images, X-ray diffraction, XPS spectroscopy, ^57^Fe Mossbauer) and electrochemical data. The calculation methodology is proposed to study the formation of M–N–C active sites, from the beginning of the thermal process, without limiting the calculations to a specific metal active site^56–59^.

### Theoretical studies

**Figure 1:**
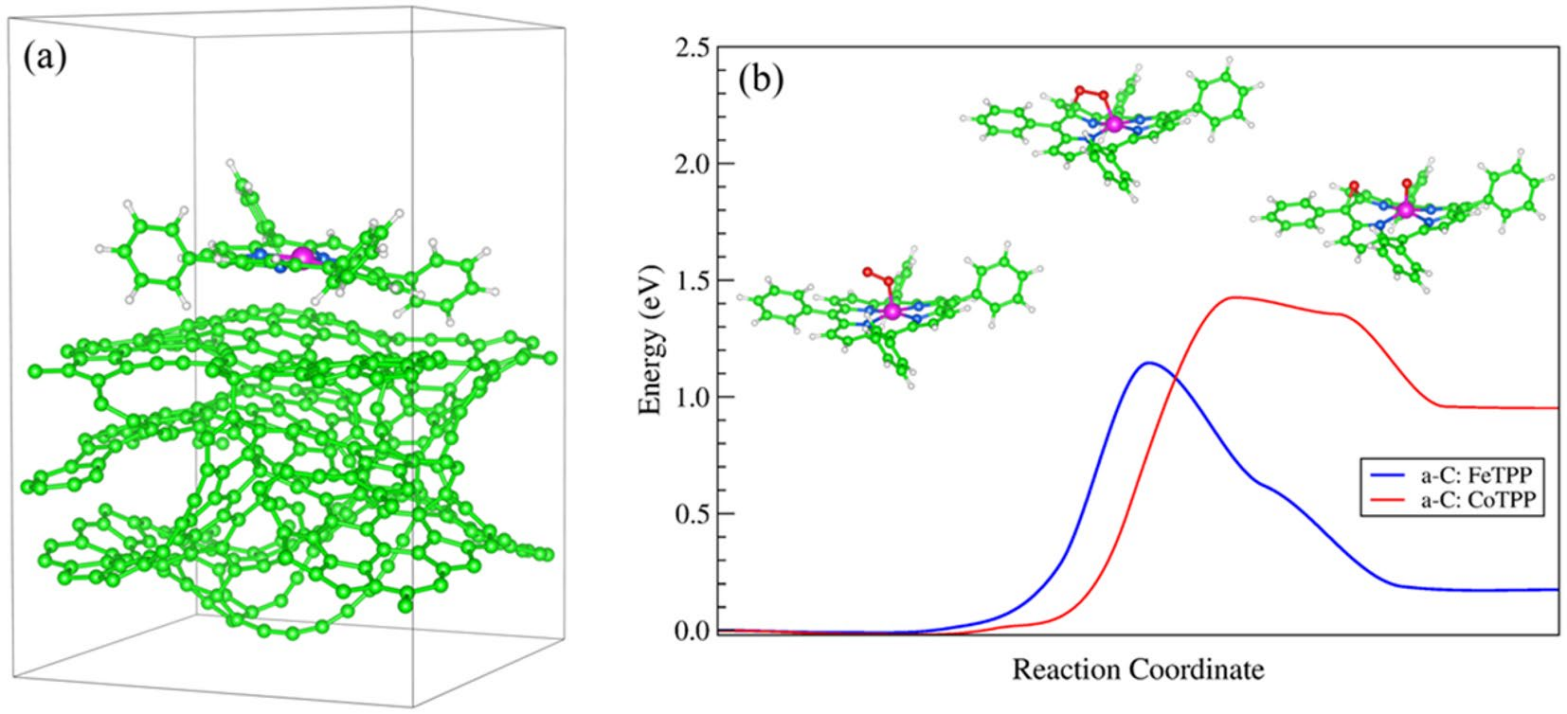
**(a)** Equilibrium geometry of the FeTPP molecule adsorbed on amorphous carbon after heat treatment. **(b)** Minimum energy path for the O_2_ dissociation after the interaction with the metal center of the FeTPP and CoTPP which are supported on *a*-C (which is removed from de inset figures for clarity). The initial, saddle point and final geometries of O_2_ interacting with FeTPP through the reaction coordinate are also shown.

Co and Fe tetraphenyl porphyrin (CoTPP and FeTPP) were selected as catalysts for the ORR because of their strong $\pi$–$\pi$ stacking interactions with the carbonaceous matrix (carbon Vulcan), used as substrate, and for the good electrocatalytic activity^60–64^. The band structure calculation shows that the amorphous carbon has metallic characteristic, as shown in supporting information Fig. S1. Figure 1a shows the equilibrium geometry of the FeTPP adsorbed on the *amorphous carbon* (*a-*C) after pyrolysis at 800 °C and subsequent optimization. The FeTPP molecule is strongly physiosorbed on the irregular *a-*C surface with binding energy of − 2.27 eV. The system shows a magnetic moment *m* = 2.24 μ_B_. Similar results were found for the CoTPP molecule on *a-*C, but with binding energy and magnetic moment of − 2.25 eV and 1.00 μ_B_, respectively. The molecules tend to preserve their spin multiplicity after the adsorption on the *a-*C substrate. However, it is observed a small spin polarization of the substrate resulting from a net magnetization higher than the isolated molecule, which are found of 0.86 and 2.06 μ_B_ for CoTPP and FeTPP, respectively.

The O_2_ interaction with the metal center of FeTPP and CoTPP supported on *a*-C were investigated. O_2_ attaches on Fe and Co atoms with binding energies of − 0.64 and − 0.48 eV, respectively, following the end-on configurations. A small reduction in the O_2_ binding energy by the action of the carbon substrate can be noticed. Details of the O_2_ attachment on the porphyrins are shown in Table 1. The spin coupling between O_2_ and the metal atoms results in a net magnetization of 0.80 and 0.72 μ_B_ for FeTPP and CoTPP, indicating a low spin configuration of the adducts. The O–O bond distance of the adsorbed O_2_ increases by around 7% on the Fe and 6% on the Co, with respect to gas phase O_2_, suggesting an ORR catalytic activity. To verify this activity, we calculated the minimum energy path and saddle point for the O_2_ dissociation starting from initial and final geometries previously optimized, using the NEB method. The results are shown in Fig. 1b. An energy barrier of 1.15 eV for the O_2_ dissociation after the interaction with the metal center of the carbon-supported FeTPP, was calculated as shown in the inset images of Fig. 1b. Similar results are found for the O_2_ dissociation on the carbon-supported CoTPP, but on this macrocycle, the energy barrier is much higher (1.42 eV). The results reveal a much higher ORR activity for the FeTPP than CoTPP, which is also better than those found on Fe Phthalocyanine supported on carbon nanotubes without pyrolysis process and previously reported^14,62^.

**Table 1:** Bond distances and binding energy of O_2_ adsorbed on the metal center of FeTPP and CoTPP molecules, free standing and supported on carbon (*a*-C).

Adduct	E_bind_ [O_2_] (eV)	d_O-O_ (Å)	d_O-M_ (Å)	d_M-N1_ (Å)	d_M-N2_ (Å)	d_M-N3_ (Å)	d_M-N4_ (Å)	*m* (μ_B_)
FeTPP-800 °C	− 0.637	1.315	1.927	2.014	2.009	2.022	1.997	0.80
CoTPP-800 °C	− 0.481	1.305	1.957	2.002	1.994	2.010	1.985	0.72
FeTPP-25 °C	− 0.680	1.320	1.908	2.019	2.014	2.009	2.000	0.68
CoTPP-25 °C	− 0.502	1.308	1.959	1.996	2.004	1.994	1.996	0.73

d_O-M_ is the distance between O and M atoms (M = Co, Fe), d_M-N_ is the distance between M and the N atoms, and *m* is the magnetic moment of the systems. The bond length of gas-phase O_2_ is calculated to be of 1.234 Å.

In order to verify the stability of the FeTPP macrocycle on *a*-C at higher pyrolysis temperature, we performed AIMD simulation at 1500 °C, during 2 ps of simulation time. The FeTPP structural stability is preserved during the simulation time, revealing the stiffness of the macrocycle. A snapshot of the *a*-C:FeTPP geometry taken at 2 ps of the AIMD simulation is shown in supporting information Fig. S2.

### Spectroscopic studies

**Figure 2:**
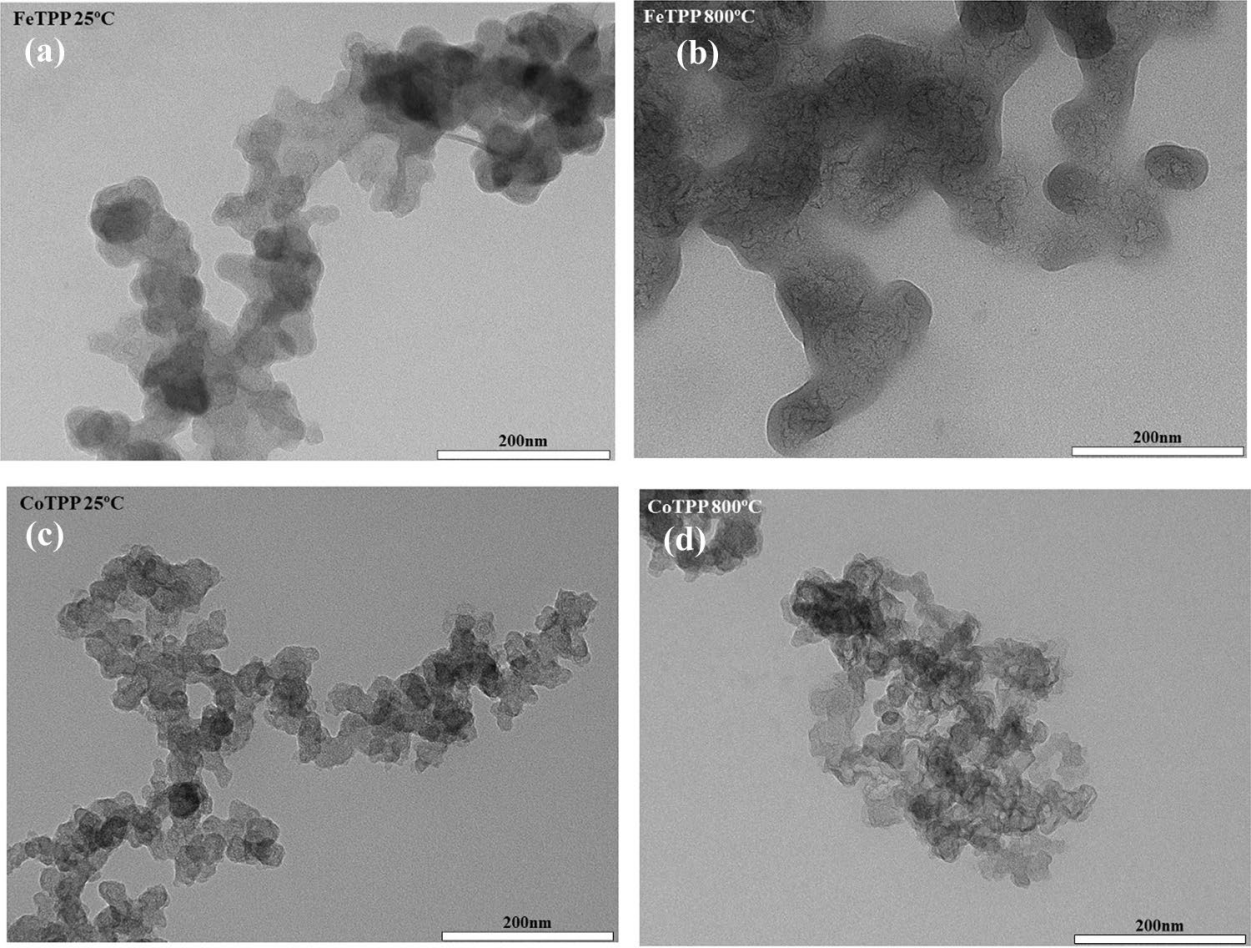
Transmission electron microscopy images of (**a**) FeTPP 25 °C, (**b**) FeTPP 800 °C, (**c**) CoTPP 25 °C, and (**d**) CoTPP 800 °C adsorbed on Vulcan XC-72.

In Fig. 2a–d we show the TEM images of the surface structure of the supporting carbon material before (Fig. 2a,c) and after (Fig. 2b,d) heat treatment. After pyrolysis a more exposed structure can be observed. Nonetheless, major structural changes are excluded. Along with this, we could not find presence of nanoparticles or crystals formed by *π-π* stacking^65–67^. This fact is also supported by X-ray diffraction (Fig. S3), where samples at 25 °C, as well as 800 °C show a characteristic peak at approximately 2*θ* = 25.4° and 43.3° associated with graphite (002) and (100) planes for attached MN4 complex^68–70^. Also, no nanoparticles diffraction peak is visualized before or after heat treatment, suggesting that the possible variation in electrocatalytic performances should not be attributed to the formation of nanoparticles or nitrides and just to MN4 sites^16,22,71–74^.

**Figure 3:**
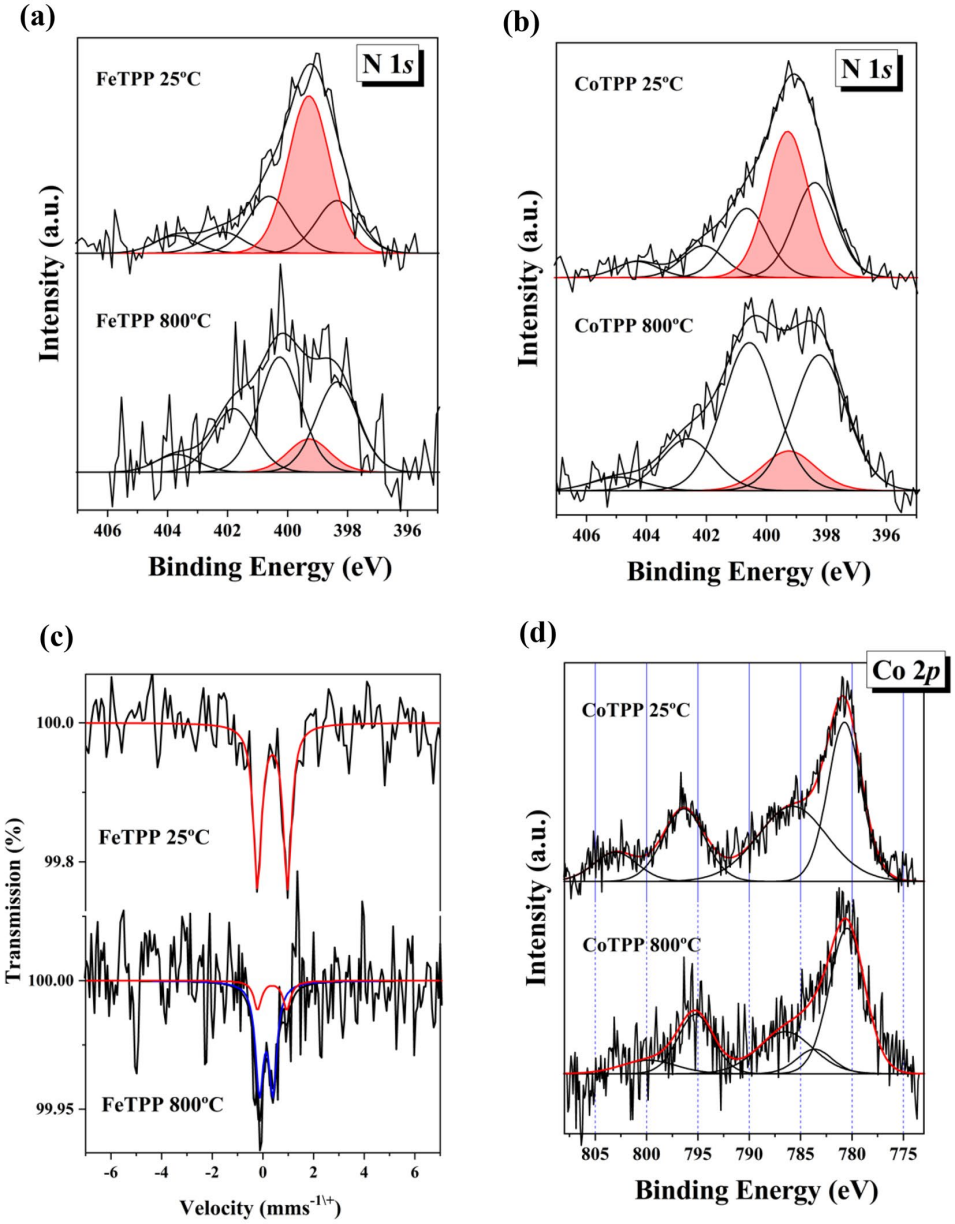
(**a**,**b**) N1s XPS spectra recorded from the FeTTP and CoTPP materials adsorbed on Vulcan XC-72 before and after pyrolysis; (**c**) room temperature ^57^Fe Mossbauer spectra recorded from the FeTPP materials; (**d**) Co 2p XPS spectra recorded from the CoTPP materials.

The samples were also characterized by XPS spectroscopy (Fig. 3a,b and Fig. S4a,b) using procedures similar to those used in previous works^22,71^. Figure 3a,b show the N 1 s spectra recorded from FeTPP (25 °C and 800 °C) and CoTPP (25 °C and 800 °C). The load of iron in the FeTPP catalysts was quite small and this precluded recording Fe 2p XPS spectra with good statistics. The spectra were computer-fitted using the constraint of equal full width at half maximum (FWHM) for all the spectral components in order to avoid undesired fluctuations in the relative obtained areas. Five contributions were used to fit adequately the experimental data which were located at the binding energies 403.9 eV (N-oxide) 402.1 eV (N-graphitic), 400.9 eV (N-pyrrolic), 398.5 eV (N-pyridinic), and, finally, at 399.9 eV (MN4 sites; M: Fe or Co) and which are usually associated in the literature with the catalytic M–N–C active site^16,22,71–73^. The relative areas of the different components and their assignment to the various chemical species are collected in Table S1 of the supplementary information. The spectra recorded from the Fe- and CoTPP 25 °C are both dominated by the MN4 contribution (Fig. 3a,b). After pyrolysis at 800 °C the intensity of this component is reduced significantly in both the iron- and cobalt materials. This might be related with a smaller concentration of iron and cobalt *(*vide infra*)* in the pyrolyzed samples as compared with the original samples which is consequently reflected in a smaller number of MN4 sites. It must be noted that the N 1 s spectra of the pyrolyzed materials have a lower signal-to-noise ratio than the corresponding of the 25 °C samples. Therefore, it seems that the pyrolysis treatment also reduces the nitrogen content of the materials. It is quite remarkable that the iron and cobalt samples show only small differences in their respective N 1 s spectra. This suggests that the preparation method of the materials before and after pyrolysis is quite reliable and the behavior almost independent of the type of metal.

To gather more information about the nature of the iron species in these samples, we resorted to Mossbauer spectroscopy (Fig. 3c). The Mossbauer spectra also showed a poor signal-to-noise ratio as a consequence of a small iron load. The absorption of the spectrum corresponding to the FeTPP 800 °C sample is much lower than that shown by the FeTPP 25 °C sample, indicating that the latter has a larger iron concentration. This supports the reduction observed in the intensity of the Fe-N4 signal in the N 1 s XPS spectra. The room temperature ^57^Fe Mössbauer spectrum recorded from FeTPP 25 °C consisted of a single, narrow paramagnetic doublet with hyperfine parameters δ = 0.38 mms^-1^, Δ = 1.18 mms^−1^. These parameters are characteristic of low spin Fe(II) in Fe^II^N4 species^39^. The result is important since (i) it shows the presence of this specie in the freshly prepared catalyst confirming the XPS results and (ii) it indicates that this is the only iron species present in this material. The Mossbauer spectrum recorded from the FeTPP 800 °C sample (Fig. 3c) is different. It shows two different paramagnetic doublets. The minor one (27% of the spectral area) has identical parameters than the doublet present in the spectrum of FeTPP 25 °C and, therefore, it corresponds to Fe^II^N4 sites. The hyperfine parameters of the major doublet (77%) δ = 0.14 mms^−1^, Δ = 0.51 mms^−1^ cannot be associated to any of the usual iron species appearing in this type of materials^39^ and resemble more those shown by some defective paramagnetic cubic iron mononitrides such as $\upgamma \prime \prime \prime$-FeN^75^. Such type of nitrides can be stabilized by rapid quenching after heating above 800 °C and, being of austenic nature, where the presence of carbon might help in this stabilization.

The Co 2p spectra from the cobalt-containing materials are shown in Fig. 3d. The signal-to-noise ratio of the Co 2p spectrum corresponding to CoTPP 800 °C sample was smaller than that of CoTPP 25 °C. This, again, suggests a smaller metal content in the pyrolyzed materials. The Co 2p spectrum recorded from CoTPP 25 °C consists of a spin orbit doublet with intense shake-up structure behind the main Co 2p_3/2_ and Co 2p_1/2_ photoemission lines. The binding energies corresponding to these core levels are 780.7 eV (Co 2p_3/2_) and 796.4 eV (Co 2p_1/2_) while the binding energies of the shake-up satellites are 785.8 eV and 803.0 eV. The structure of the spectrum and the binding energies of the main core level lines, with a spin–orbit energy separation of 15.7 eV, are characteristic of a high spin S = 3/2 Co(II) species^76,77^. The Co 2p spectrum recorded from the CoTPP 800 °C, looks different. It has a less prominent shake up structure appearing at 786.5 eV and 799.8 eV (the latter satellite being very weak), a multiplet splitting component at 783.6 eV and main photoemission lines appearing at the binding energies of 780.5 eV (Co 2p_3/2_) and 795.2 eV (Co 2p_3/2_), i.e., with a spin–orbit splitting energy of 14.7 eV (1 eV smaller than in the other sample). All these data together can be associated with the presence of an intermediate spin S = 1 Co(III) species^13^.

Resuming, the spectroscopic results show that the amorphous carbon structure is not significantly altered by the heat treatment and that the FeTPP and the CoTPP are mainly incorporated to the carbon support by strong *π-π* stacking with an N-M bonds length which might become slightly larger after pyrolysis as in accordance to the DFT calculations (Table 1) and to a more exposed structure as observed in TEM images (Fig. 2). The concentration of Fe and Co decreases during the pyrolysis process accompanied with the formation of nitrides.

### Electrochemical studies

**Figure 4:**
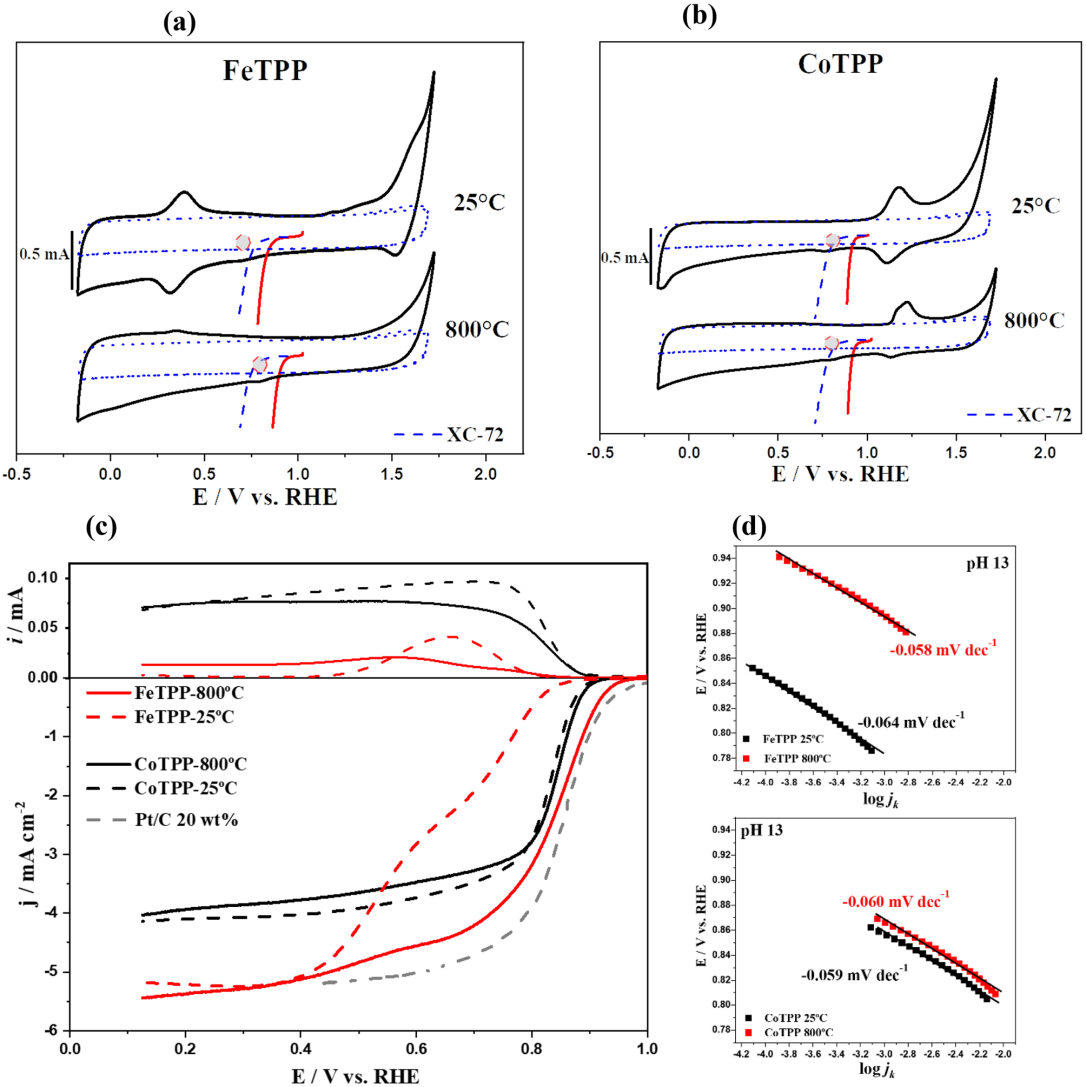
Cyclic voltammetry at 100 mV s^−1^ in N_2_ saturated atmosphere (black line) and polarization curve *i*_*L*_ corrected [(*i*_L*_i)/(*i*_*L*_*-i*)] in O_2_ saturated atmosphere (red line) with 0.1 M NaOH as electrolytic solution for **(a)** FeTPP, FeTPP-800 °C, and Vulcan XC-72 **(b)** CoTPP, CoTPP-800 °C, and Vulcan XC-72. **(c)** Ring-disk current curves for linear sweep voltammetry at 5 mV s^−1^ in 0.1 M NaOH O_2_ saturated atmosphere for FeTPP or CoTPP at 25(dash line) and after pyrolysis at 800 °C (CoTPP-800 °C, FeTPP-800 °C, solid line), and commercial Pt/C 20 wt% (gray dash line) as from Fig. S5. **(d)** Tafel plot for the ORR for FeTPP and CoTPP before and after pyrolysis at 800 °C.

Figure 4a,b shows cyclic voltammetry in N_2_ atmosphere together with their respective linear sweep voltammetry (LSV, at 1600 rpm) curves in O_2_ saturated buffer for pyrolyzed and unpyrolyzed FeTPP and CoTPP, and Vulcan XC-72 in alkaline media, Fig. 4c. All the obtained values are summarized in Table 2. To determine the Tafel plot in Fig. 4d, the LSV was corrected by the limiting current contribution [*j*_L_* j]*/*[*j*_*L*_*-j*]^78^ before using Butler–Volmer equation^78^ and to avoid diffusional contribution (*j*_*L*_) in the kinetic electron-transfer process (*j*_*k*_). The M(III)/(II) formal potential has been detected by cyclic voltammetry and is shown with a grey circle in Fig. 4a,b. The values are in agreement with those reported in the literature^16,22,71^. As reported in Table 2 and Fig. 4a,b, and graphical abstract, there is a connection between the formal potential of the M(III)/(II) redox couple and the onset potential (*E*_onset_) for the ORR, and an increase in the catalytic current^79^. This can be noticed for all the catalysts (i.e., before and after the heat treatment) and has been widely reported for monolayers of porphyrins and phthalocyanines as well as for pyrolyzed complexes^10,16,80^. Further, from the electrochemical results, two facts can be observed (*i*) for non-pyrolyzed Co and Fe porphyrins, the CoTPP shows higher catalytic activity than FeTPP (Fig. 4c, dash black and red line respectively) in agreement with the lower ionization potential of Fe-porphyrins which make them worse catalysts than cobalt counterpart^16,81–83^. (*ii*) There is a shift of *E*^0’^ for Co(III)/Co(II) and Fe(III)/Fe(II) to more positive values after pyrolysis, which indicates an environmental electronic modification that affects the metal redox centers and the catalytic activity, where the M(II) sites are required for the ORR process to take place in basic media as for an inner-sphere electron transfer mechanism (ISET)^71,84,85^. Several authors affirm that changes in the nature of carbonaceous support matrix as graphitic/amorphous structure or surface defects after the pyrolysis could be responsible for changes in catalytic activity toward the ORR with intrinsic modification in spin distribution for the carbon matrix^86–89^. This is in agreement with the herein reported DFT simulations. The electrons transferred during the ORR were calculated with the rotating ring-disk electrode (Eq. (3))^78^. Results are resumed in Table 2 and show numbers close to 3 and 4 electrons transferred by CoTPP and FeTPP respectively. A lower percentage of hydrogen peroxide is produced by FeTPP, before and after heat treatment, compared to CoTPP in similar conditions (values are resumed in Table 2). These results indicated direct H_2_O formation or 2 + 2 mechanism for FeTPP^90^, while for CoTPP water and peroxide formation are simultaneously formed during the reduction mechanism. FeTPP, after pyrolysis, showed better performance for the ORR than CoTPP, as we expected from DFT calculations of the energy barrier for the O_2_ dissociation in Fig. 1b, because of the lower interplay between O_2_ and Co metal center in contrast to Fe centers (see Table 1). In Fig. 4d, similar Tafel slope values for Co and Fe porphyrins before and after pyrolysis are reported. The value is close to − 0.060 V dec^−1^ at lower overpotential, where M(II) concentrations are depending on the applied potential^22,91^. The Tafel slope value indicates that the rate-determining step (r.d.s.) is chemical O_2_ adsorption in M(II) preceded by a fast first electron transfer for ISET mechanism, which is a common step in the studied catalysts^22,87,92^ according to the following equation:

1 $$\left[ {M\left( {III} \right) - OH^{ - } } \right]_{ad} + H_{2} O + e^{ - } \rightleftarrows { }\left[ {M\left( {II} \right) - OH_{2} } \right]_{ad} + { }OH^{ - } { }$$

2 $$\left[ {M\left( {II} \right) - OH_{2} } \right]_{ad} \,+\, O_{2}\, + \,e^{ - } \to \left[ {M\left( {III} \right) - O_{2} } \right]_{ad}^{ - } \,+\, H_{2} O \rightleftarrows \left[ {M\left( {II} \right) - OH_{2} } \right]_{ad} \,+ \,H_{2} O\, \left( {{\text{r}}.{\text{d}}.{\text{s}}. \, - 0.0{6}0{\text{ V dec}}^{{ - {1}}} } \right)$$

**Table 2:** Electrochemical parameter for pyrolyzed and non-pyrolyzed catalysts.

	Formal potential [V vs. RHE]	Onset [V vs. RHE]	Tafel (V/dec)	Ring-Disk	Peroxide at 0.3 V vs. RHE (%)
CoTPP-pyrolyzed	0.819 ± 4 × 10^–3^	0.883 ± 1 × 10^–3^	− 0.060 ± 3 × 10^–4^	3.1 ± 5 × 10^–2^	53.7
FeTPP-pyrolyzed	0.841 ± 9 × 10^–3^	0.930 ± 2. × 10^–3^	− 0.058 ± 1 × 10^–3^	4.0 ± 7 × 10^–2^	3.2
CoTPP-non-pyrolyzed	0.792 ± 7 × 10^–3^	0.870 ± 4 × 10^–4^	− 0.059 ± 2 × 10^–4^	3.0 ± 6 × 10^–2^	54.1
FeTPP-non-pyrolyzed	0.705 ± 4 × 10^–3^	0.830 ± 6 × 10^–3^	− 0.064 ± 1 × 10^–3^	3.9 ± 8 × 10^–2^	1.1

In light of these results (values resumed in Table 2), there is no significant variation in the ORR mechanism before and after heat treatment, suggesting the similar nature of the M–N–C active site after pyrolysis. Nevertheless, the structure of the resulting catalysts is enormously depending on the synthesis route and pyrolysis process^93–96^. We attribute the enhancement in the catalytic activity for the ORR to variations in the interactions with the Vulcan substrate, which induces fluctuations in the electron density of the metal redox center, causing changes to the binding energy between M-O_2_ as reported in Table 1. This also relates with the differences in the M–N bond length in the catalyst structure after pyrolysis. Finally, if we consider the catalytic activity as log *j*_*k*_ at constant potential (*E*: 0.8 V vs. RHE) versus binding energy M-O_2_ and also versus the formal potential, as proposed by several authors^10,16,61,97–99^, we obtain the figure reported as graphical abstract, where the MN4 structure is maintained, and the catalysts follow the trend of the correlations.

## Conclusions

Ab initio molecular dynamic simulations were carried out for the pyrolysis of CoTTP and FeTPP adsorbed on amorphous carbon. The theoretical results indicated that there is no breakdown of the macrocyclic complex at elevated temperatures, up to 1500 °C, preserving the MN4 geometry. However, variations in the M-O_2_ binding energies were observed after the simulating pyrolysis at 800 °C, which is attributed to the dispersive interaction between the macrocycles and the irregular surface of the *a*-C substrate, inducing small variation in the MN4 bond distance. TEM images showed a more exposed structure after the pyrolytic process. XRD measurements excluded the presence of nanoparticles or MN4 stacks or crystals before or after the pyrolysis. The XPS results corroborate the presence of MNx catalytic sites for both complexes, before and after heat treatment. Through electrochemical studies we could detect a displacement of the formal potential to more positive values after the thermal treatment, but the Tafel slopes did not vary showing that the ORR mechanism did not change before and after the pyrolysis. It is suggested that the variation in the catalytic activity for the ORR is associated with the variations in the interaction of Fe and Co porphyrins with the carbon substrate, where the porphyrinic M–N–C active site is preserved after pyrolysis. If it is desired to introduce nitrogen and metal atoms into the carbon structure, other strategies have to be adopted, such as the solid-state synthesis with metal salts and carbon–nitrogen sources instead of using metallo-macro-cycles. The DFT calculations proved to be a powerful tool to simulate the changes of M–N–C active sites during pyrolytic processes.

## Materials and methods

### Catalysts preparation and synthesis

Fe(III) 5,10,15,20-(tetraphenyl)porphyrin chloride and Co(III) 5,10,15,20-(tetraphenyl)porphyrin chloride were supplied by *PorphyChem* (Dijon, France). Both metalloporphyrins were mixed separately with Vulcan XC-72® in agata mortar for 40 min (MN4:XC-72; 1:4 mass ratio), then 1 mg of catalyst powder was dispersed in 1 mL of isopropanol: water (1:4). The resulting ink was stirred for 5 h, then dried at 60 °C for 5 h. The first fraction of obtained powder was denoted as FeTPP-25 °C and CoTPP-25 °C. A second fraction was pyrolyzed under N_2_ atmosphere at 800 °C, carried out in a Furnace 1200 °C (SK-G03123K). The pyrolyzed samples were labelled FeTPP-800 °C and CoTPP-800 °C, respectively. Finally, the working electrode was modified by a spin coating process, dropping 10 µL of ink (Co or FeTTP at 25 or 800 °C) containing 1 mg of powder catalyst in 1 mL of isopropanol:water, 1:4, and 0.7 Vol% of 5% Nafion® solution, to final surface concentration of 0.05 mg cm^−2^ of the catalysts. Pt/C 20 wt% and Vulcan XC-72 (FuellCell) ink was obtained using a similar procedure as for the FeTPP-25 °C and CoTPP-25 °C catalysts.

### Theoretical simulations

Spin-polarized density functional theory (DFT) calculations were performed using the Quantum Espresso ab initio package^100^. Dispersive interactions were included by the non-local van der Waals correlations into the exchange correlation potential (vdW-DF2)^101–105^. Kohn–Sham eigenfunctions are expanded on a plane-waves basis set where the interaction between valence electrons and ion cores are described by ultrasoft pseudopotentials^106^. Converged results have been achieved by cut off energies of 480 eV. All atoms are left free to relax until the residual force on each atomic component was less than 0.002 eV/Å. The sampling of the Brillouin zone was performed with the Γ point. The minimum energy path for the O_2_ dissociation was obtained using the climbing Nudged Elastic Band (NEB) method^107,108^, using 8 images throughout the reaction coordinate. Amorphous carbon was obtained from the diamond structure through ab initio molecular dynamic (AIMD) simulation, using the Vienna Ab Initio Simulation Package (VASP)^109^. Here we employ the generalized gradient approximation for the exchange–correlation functional and a cut off energy of 400 eV for the plane-wave basis set. The core-valence interaction was described by the projector augmented-wave method^110^. A large periodic unit cell of 17.8 × 17.8 × 16.0 Å^3^ containing a diamond slab with 7 monolayers was thermalized at 4000 K during a simulation time of 5 ps and then cooled down to 0 K during 2 ps, using a time step of 1 fs. The resulting structure was then optimized by self-consistent DFT calculations after the equilibrium geometry is achieved. Pyrolyzed FeTPP and CoTPP adsorbed on the amorphous carbon structure was obtained by AIMD simulation. The system was thermalized at 1073 K during a simulation time of 2 ps and then cooled down to 0 K during 2 ps.

### Spectroscopic characterization

The transmission electronic microscopy (TEM) was performed by depositing the ink on the TEM grid until it dried. The TEM images were obtained using Hitachi HT7700 equipment with an accelerating voltage of 120 kV.

X-ray diffraction (XRD) was used to determine the nanoparticle presence before and after-heat treatment, performed in Bruker D8 Advance diffractometer with CuK_α1,2_ radiation (λ = 1.54118 Å) at 40 mA and 40 kV.

X-ray photoelectron spectroscopy data were recorded under a vacuum better than 2.0 × 10^–9^ mbar using a PHOIBOS-150 electron analyzer (SPECS), with a constant pass energy of 20 eV and Al Kα radiation. The binding energy scale was referenced to the main C–C C1s signal located at 284.6 eV.

^57^Fe Mossbauer spectra were recorded from the iron-containing samples. The data were taken in the transmission mode at room temperature using a conventional constant acceleration spectrometer. The velocity scale was calibrated using a natural alpha-iron foil 6 μm thick. The spectra were computer-fitted and the isomer shifts referred to the centroid of the sextet of alpha-iron at room temperature.

### Electrochemical characterization

A typical three electrodes electrochemical cell was used during the electrochemical experiments, using *Hg*^*0*^*/Hg*_*2*_*Cl*_*2*_ calomel electrode and *carbon stick* as reference and counter electrode respectively. Edge-plane pyrolytic graphite disk mounted into a Teflon rotating shaft containing the *Pt*^*0*^ ring as working electrode. The disk was 5 mm diameter and 4 mm thick, while the ring diameter was 6.5 mm and the external diameter of the ring was 7.50 mm. The rotor system for the -ring-disk electrode was from PINE Instrument (Durham, NC, USA). Sodium hydroxide from Merck Millipore (NaOH, ≥ 99%) was used to prepare 0.1 M NaOH as electrolytic solution with Milli-Q water (18.2 MΩ·cm at 25 °C). Cyclic voltammetry characterization was done at 100 mV s^−1^ to observe faradic processes using 0.1 M NaOH electrolytic solution and N_2_ saturated atmosphere. The electrocatalytic activity was studied at 5 mV s^−1^ in O_2_ saturated atmosphere, using an Autolab PGSTAT 204 bipotentiostat. The electrons transferred and the peroxide formation percentage during the ORR was determined by rotating -ring -disk using Eqs. (3) and (4) respectively^78,111,112^:

3 $$n_{{e^{ - } }} = \frac{{4I_{D} }}{{I_{D} + (I_{R} /N)}}$$

4 $$\% HO_{2}^{ - } = 200 \times \frac{{I_{R} /N}}{{I_{D} + (I_{R} /N)}}$$

where *I*_R_ = ring current, *I*_D_ = ring current; *N* = collection efficiency (0.39). Finally, Tafel slopes were obtained by Butler–Volmer equation^78^ to derivate the determining rate step in each catalyst.

## Supplementary Information

Supplementary Information.

## Data Availability

The data generated and analysed during the current study are available from the corresponding author.
