# Supplementary material for: Evidence of carbon-supported porphyrins pyrolyzed for the oxygen reduction reaction keeping integrity
Source: Sci Rep. 2022 May 16;12:8072. doi: 10.1038/s41598-022-11820-6 (PMC9110719; doi:10.1038/s41598-022-11820-6)
Supplement: Supplementary file 1 — Supplementary Information. [file 41598_2022_11820_MOESM1_ESM.docx]

**Supporting Information**

**Evidence of Carbon-Supported Porphyrins Pyrolyzed for the Oxygen Reduction Reaction Keeping Integrity**

Walter Orellana^a^, César Zúñiga Loyola^b*^, José F. Marco^c^, F. Tasca^b*^

*^a^Departamento de Ciencias Físicas, Universidad Andrés Bello, Sazié 2212, 837-0136 Santiago, Chile.*

*^b^ Departamento de Química de Los Materiales, Facultad de Química y Biología, Universidad de Santiago de Chile, Santiago, Chile.*

*^c^Instituto de Química Física “Rocasolano” CSIC, Spain.*

**Corresponding author:* *[federico.tasca@usach.cl](mailto:federico.tasca@usach.cl),* [*cesar.zunigal@usach.cl*](mailto:cesar.zunigal@usach.cl)

**
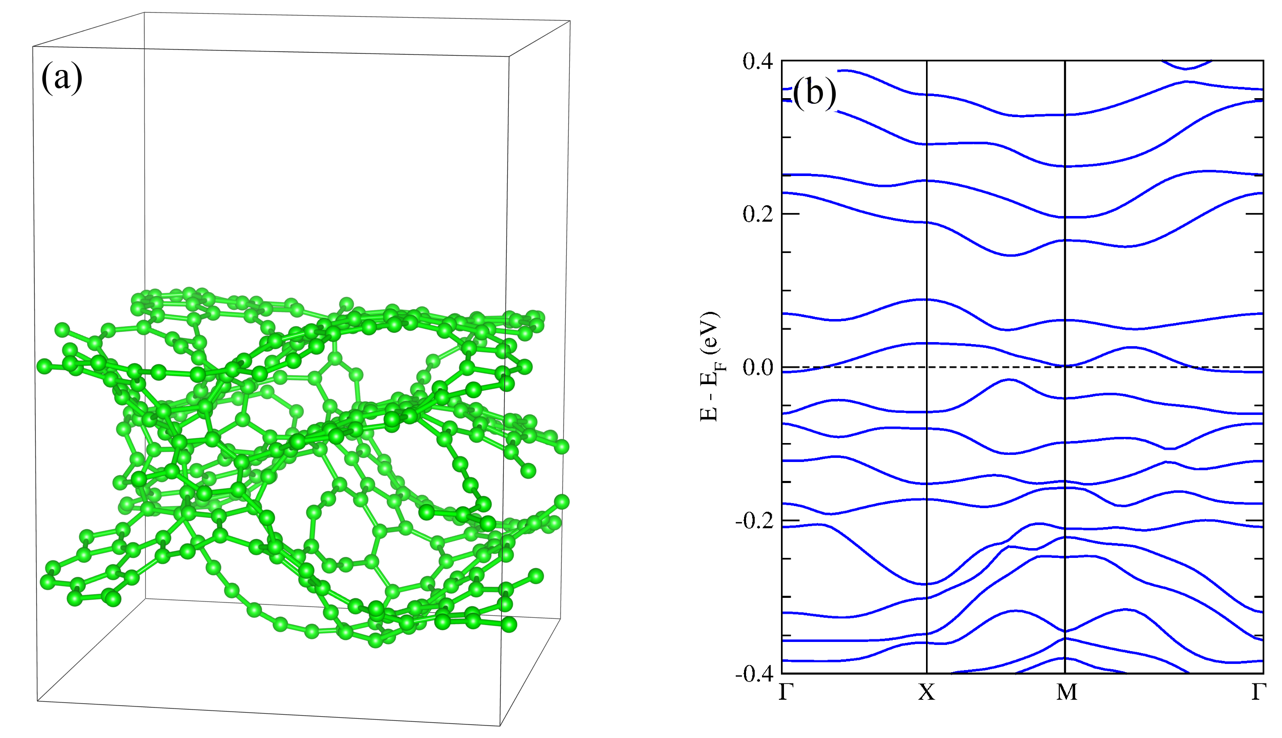
**

**Figure S1: (a)** Equilibrium geometry of the amorphous carbon (*a*-C) structure as obtained from AIMD simulations at 4000K and later optimization at 0K by self-consistent DFT calculations in a periodic unit cell. **(b)** Band structure calculation of the amorphous carbon at the equilibrium geometry.


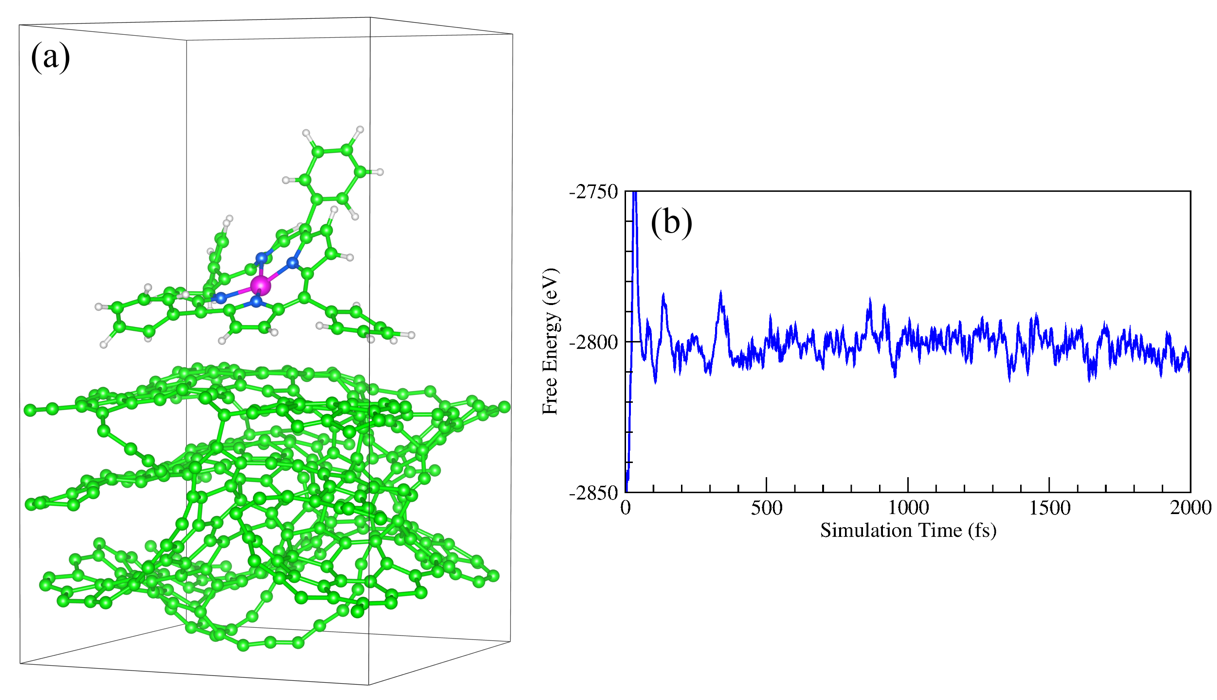


**Figure S2: (a)** Snapshot of the *a*-C:FeTPP geometry taken at 2 ps of AIMD simulation at 1500 ºC. **(b)** Free energy evolution as a function of the simulation time for the AIMD simulation. After 2 ps the FeTPP molecule preserves its integrity, indicating that pyrolysis process at lower temperatures would not induce the macrocycle breakdown.


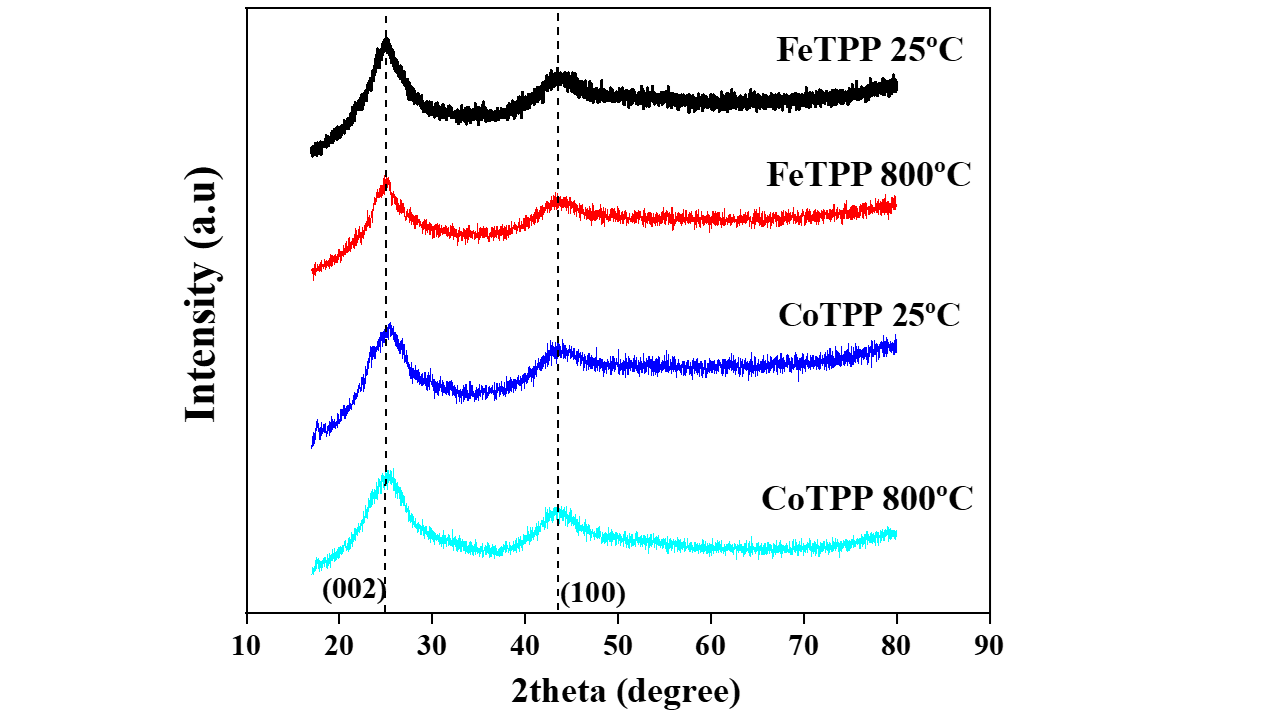


**Figure S3:** X-diffraction pattern (XRD) for FeTPP and CoTPP catalysts measured before and after pyrolysis treatment.


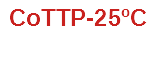

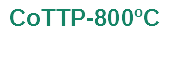

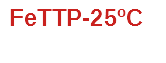

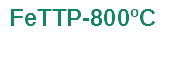


**(a)**

**(b)**

**Figure S4:** Wide scan XPS spectra recorded for (a) FeTPP and (b) CoTPP at 25ºC and 800ºC


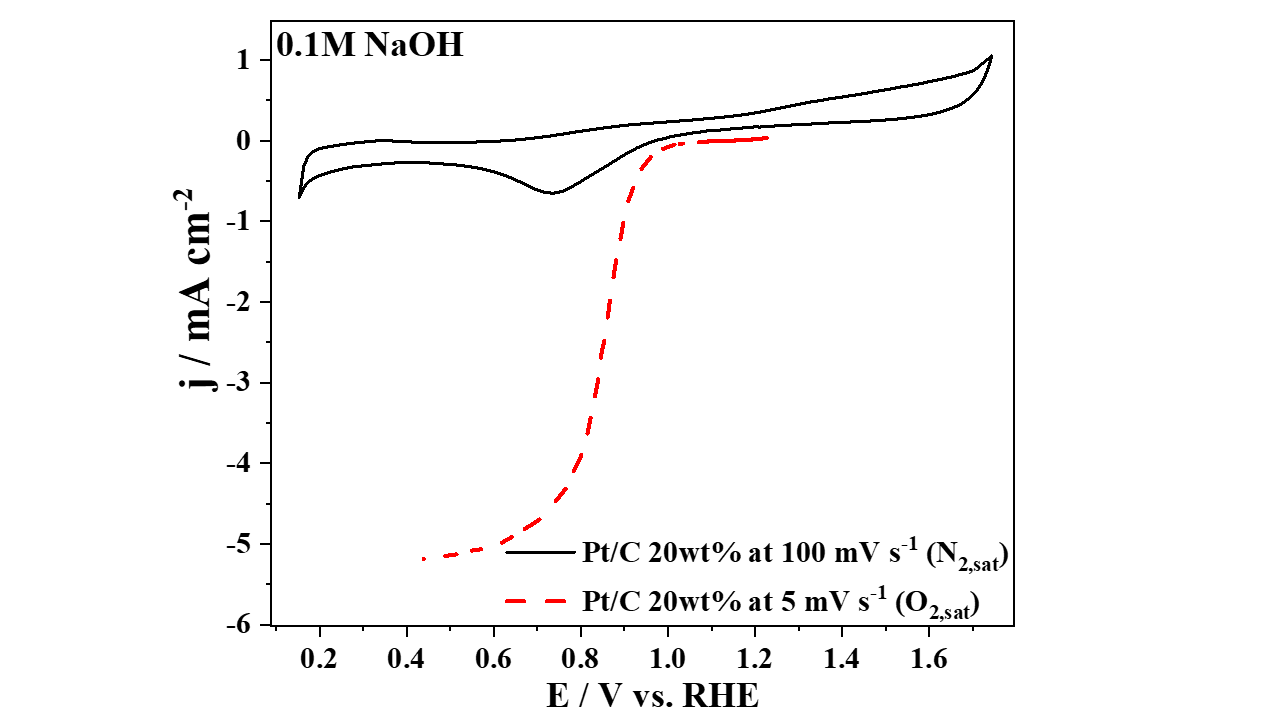


**Figure S5:** Cyclic voltammetry (black line) and polarization curve (red line) at 100 mV s^-1^ and 5 mV s^-1^ respectively, in N_2_ or O_2_ saturated atmosphere from reference^1^.

**Table S1**. Relative areas of the different nitrogen contributions to the N 1s spectra recorded from the various materials studied in this work.

|  | **FeTPP 25ºC** | **FeTPP 800ºC** | **CoTPP 25ºC** | **CoTPP 800ºC** |
| --- | --- | --- | --- | --- |
| **Pyridinic (%)** | 17 | 28 | 27 | 35 |
| **N-Fe (%)** | 52 | 10 | 41 | 10 |
| **Pyrrolic (%)** | 19 | 36 | 19 | 38 |
| **Graphitic (%)** | 7 | 20 | 9 | 13 |
| **N-O_x._ (%)** | 6 | 6 | 5 | 3 |

**References**

1. Loyola, C. Z. *et al.* Insights into the electronic structure of Fe penta-coordinated complexes. Spectroscopic examination and electrochemical analysis for the oxygen reduction and oxygen evolution reactions. *Journal of Materials Chemistry A* **9**, 23802–23816 (2021).
